# Podcast Listening, Perceived Social Presence, Perceived Social Support, and Subjective Well-Being Among Chinese Young Adults: Sequential Explanatory Mixed Methods Study

**DOI:** 10.3390/bs16020267

**Published:** 2026-02-11

**Authors:** Weiwei Li

**Affiliations:** School of Philosophy and Social Development, Shandong University, Jinan 250100, China; 202390000132@sdu.edu.cn; Tel.: +86-13-682-004-911

**Keywords:** podcast, young adults, subjective well-being, perceived social presence, perceived social support

## Abstract

Background: Podcasts have emerged as a prominent audio medium in the everyday lives of young adults. Despite their growing popularity, the relationship between podcast listening and subjective well-being, along with the psychological mechanisms underlying this association, remains underexplored. This study investigates the relationship between podcast listening and subjective well-being among Chinese young adults and examines the serial mediating effects of perceived social presence and perceived social support. In doing so, it seeks to clarify how immediate media-related experiences are translated into more stable psychological resources that promote mental health. Methods: A sequential explanatory mixed-methods approach was employed. The quantitative phase involved a questionnaire-based survey of 357 participants, measuring podcast listening behavior, perceived social presence, perceived social support, and subjective well-being. Serial mediation analysis was conducted to test the hypothesized indirect pathways. The qualitative phase comprised semi-structured interviews with 20 participants, and thematic analysis was used to complement and contextualize the quantitative results by exploring young listeners’ subjective psychological experiences during podcast engagement. Results: Quantitative findings revealed a significant positive association between podcast listening and subjective well-being among young adults. Both perceived social presence and perceived social support were found to mediate this relationship, constituting a statistically significant serial mediation pathway. Consistent with these results, the qualitative analysis indicated that auditory immersion in podcast listening is associated with a stronger sense of perceived social presence, characterized by feelings of companionship and parasocial interaction. This heightened sense may be internalized as perceived social support at both informational and emotional levels, and is linked to higher subjective well-being. Conclusions: The findings demonstrate that podcasts are not merely channels for information dissemination but function as audio media with meaningful psychosocial value. By identifying the serial mediating roles of perceived social presence and perceived social support, this study extends existing theoretical frameworks to the context of audio media and offers novel empirical evidence regarding the links between digital media experiences and subjective well-being among young adults.

## 1. Introduction

In the context of rapid social transformation, Chinese young adults face mounting academic pressure, intensified competition in the labor market, and escalating societal expectations (Zhan et al., 2021; Law et al., 2020). These challenges have drawn growing scholarly attention to their subjective well-being, a core indicator of mental health and quality of life (Zhao et al., 2020). Understanding how young adults maintain and enhance subjective well-being under such conditions, therefore, remains an important theoretical and practical concern.

Digital media have increasingly been recognized as a key factor shaping young adults’ subjective well-being, given their capacity to satisfy informational, social, and psychological needs (Ahn & Shin, 2013; Ma et al., 2018). Existing research, however, has focused predominantly on visually oriented platforms such as social networking sites and short-form video applications, with comparatively little attention paid to audio-based media (Liu & Chen, 2023). Prior studies on audio media have largely examined music listening as a form of emotional regulation (Loepthien & Leipold, 2022) or the role of community radio in fostering belonging (Meadows & Foxwell, 2011), leaving the psychological mechanisms linking newer forms of audio media use to subjective well-being underexplored.

Among the diverse forms of audio media, podcasts are characterized by episodic, voice-centered content that can be accessed on demand across a wide range of everyday contexts (Rime et al., 2022). In China, podcasts have become an increasingly prominent media form among young audiences; by 2025, the podcast audience exceeded 150 million, with young adults constituting the core listener base (Chinese Podcast Association, 2025). The podcast ecosystem comprises both large commercial platforms (e.g., Himalaya) and niche community platforms (e.g., Xiao Yu Zhou), supporting diverse creators from independent producers to professional media organizations. Popular Chinese podcasts often focus on personal growth, social issues, and emotional narratives, cultivating a distinctive audio space in which young listeners seek resonance, self-reflection, and emotional support amid daily pressures (Zhu & Yang, 2025). Compared with visual media, these features may be particularly relevant to young adults’ emotional experiences, offering an analytical lens for examining the psychological mechanisms through which audio media consumption may be associated with subjective well-being.

Theoretically, media effects on subjective well-being are more likely to operate through indirect psychological processes than through direct causal pathways (Zhang et al., 2023). In the context of podcast consumption, existing studies have primarily focused on audience motivations or content preferences (Tobin & Guadagno, 2022; Xu et al., 2025), resulting in two key limitations. First, much of this work remains descriptive and lacks integrative psychological models explaining how podcast engagement contributes to well-being. Second, while podcast listening often generates immediate experiences such as feeling accompanied or emotionally attuned, subjective well-being is typically supported by more enduring psychological resources (Ostic et al., 2021). The processes through which momentary listening experiences are internalized and transformed into stable well-being outcomes, therefore, warrant closer examination.

To address these gaps, this study draws on Uses and Gratifications theory (Katz et al., 1973) and proposes a serial mediation model linking podcast listening to subjective well-being through perceived social presence and perceived social support. Perceived social presence refers to individuals’ subjective sense of connection and immediacy with others in a mediated environment (Short et al., 1976; Hassanein & Head, 2007), whereas perceived social support reflects generalized beliefs that care and assistance are available when needed (Gülaçtı, 2010). Conceptually, perceived social presence represents a situational, state-like experience, while perceived social support constitutes a more durable psychological resource. Integrating these constructs provides a theoretical bridge for explaining how transient media experiences may contribute to sustained subjective well-being.

This study employs a sequential explanatory mixed-methods design (Ivankova et al., 2006). Quantitative analyses test the proposed mediation paths, while qualitative interviews further illuminate listeners’ lived experiences and the mechanisms underlying the statistical associations. By clarifying the psychological processes linking podcast listening to subjective well-being, this study contributes to a more nuanced understanding of the psychosocial effects of audio media among young adults.

## 2. Literature Review and Hypotheses

### 2.1. The Association Between Podcast Listening and Subjective Well-Being

Subjective well-being, a core construct within positive psychology, refers to individuals’ subjective evaluations of their overall quality of life. It is commonly conceptualized as comprising three interrelated components: life satisfaction, positive affect, and negative affect (Diener et al., 1999). A substantial body of research has established a consistent association between media use and subjective well-being (Weinberg & Joseph, 2017).

Within the growing literature on audio media, podcasts have been increasingly examined for their potential psychological effects. Empirical studies suggest that, relative to visually oriented media, podcasts are well suited to meeting users’ informational, emotional, and social needs, thereby creating favorable conditions for psychological well-being (Swiatek, 2018; Tobin & Guadagno, 2022). From a Uses and Gratifications perspective, sustained engagement with podcast content may facilitate emotional regulation, perceived companionship, and a sense of social connection, all of which are relevant to subjective well-being outcomes (Ruggiero, 2000; Dreer, 2021).

Evidence from applied contexts further supports the potential benefits of podcast use. In educational settings, podcasts have been shown to enhance motivation and engagement while reducing anxiety (McKinney & Page, 2009). In health promotion and psychological intervention contexts, podcast-based psychoeducational programs have been found to alleviate burnout and emotional exhaustion and to strengthen self-esteem and self-efficacy (J. L. Li et al., 2025; Carrotte et al., 2023). Together, these findings indicate that podcasts may contribute to subjective well-being through both affective and cognitive pathways.

At the same time, prior research has noted heterogeneity in podcast-related outcomes. Exposure to highly negative or conflict-oriented content may elicit short-term emotional distress (Worms-Ehrminger et al., 2024), while excessive or immersive use that substitutes for offline social interaction may generate unintended consequences, such as echo chamber effects (Rae, 2023). Despite these potential variations, the present study focuses on the overall association between podcast listening and subjective well-being.

Given that podcast use is a multidimensional construct encompassing listening intensity, usage patterns, and content preferences, this study concentrates on listening intensity as a key behavioral indicator. From the perspective of Uses and Gratifications theory, sustained engagement with media content is a necessary condition for need satisfaction and subsequent psychological effects (Ruggiero, 2000). Accordingly, listening intensity represents a theoretically grounded and empirically relevant measure of podcast consumption. The following hypothesis is proposed:

**H1.**  *Podcast listening is positively associated with young adults’ subjective well-being.*

### 2.2. The Mediating Role of Perceived Social Presence

Perceived social presence has long been theorized as a central psychological mechanism through which mediated communication produces social and emotional effects (Short et al., 1976). It is defined as individuals’ subjective sense of being socially connected with others during mediated interaction, capturing feelings of intimacy, immediacy, and authenticity (Rodgers, 2023), as well as the extent to which mediated others are experienced as socially real rather than abstract information sources (Hassanein & Head, 2007).

From a cue-based perspective, perceived social presence is shaped by the availability and salience of social signals embedded in a medium (Mayer et al., 2003). Auditory cues, particularly the human voice, convey rich affective and interpersonal information through tone, rhythm, and expressiveness, thereby reducing perceived psychological distance and enhancing interpersonal vividness (Nass & Brave, 2005). Compared with text- or image-based media, audio communication places greater emphasis on these vocal signals, making it especially conducive to eliciting perceived social presence (Bottomley, 2020).

In the context of podcast listening, the production and reception of perceived social presence can be further understood through parasocial interaction theory. Podcasts typically employ conversational narratives and informal communicative styles, which foster a sense of perceived interpersonal connection despite the unidirectional nature of the interaction (Horton & Wohl, 1956). Through repeated exposure, listeners may experience an enduring sense of asynchronous companionship with podcast hosts, a phenomenon widely recognized as a core manifestation of perceived social presence in mediated environments (Vilceanu, 2025).

Beyond shaping momentary media experiences, perceived social presence is theorized to play an important role in psychological well-being. By attenuating feelings of social distance and enhancing perceived connectedness, perceived social presence can satisfy fundamental social needs and provide a sense of relational embeddedness, even in the absence of direct interpersonal interaction (Kang & Gretzel, 2012). Prior research across mediated contexts has consistently shown that higher levels of perceived social presence are associated with greater emotional engagement, satisfaction, and overall subjective well-being, underscoring its function as a psychological bridge between media exposure and well-being outcomes (Soto-Vásquez et al., 2022). Taken together, these theoretical arguments suggest that podcast listening may foster perceived social presence, which in turn contributes to subjective well-being. Accordingly, the following hypothesis is proposed:

**H2.**  *Perceived social presence mediates the relationship between podcast listening and subjective well-being among young adults.*

### 2.3. The Mediating Role of Perceived Social Support

Perceived social support refers to individuals’ subjective beliefs regarding the availability of care, assistance, and resources within their social environment (Procidano & Heller, 1983). Unlike perceived social presence, which emphasizes the immediate experience of psychological closeness during mediated encounters, perceived social support focuses on individuals’ evaluations of whether reliable support is accessible when needed. The research demonstrates that such subjective appraisals are more powerful predictors of psychological adjustment, stress coping, and subjective well-being than the actual receipt of support (Eagle et al., 2018).

Perceived social support is commonly understood to encompass two interrelated dimensions: informational support and emotional support. Informational support involves the belief that advice, guidance, and problem-solving resources are available, whereas emotional support reflects perceived understanding, care, and emotional validation from others (Bol et al., 2022). Within the context of podcast listening, perceived informational support is primarily generated through content-based mechanisms. As an audio medium rich in knowledge and experiential narratives, podcasts often provide interpretive frameworks, practical advice, and shared experiences related to domains such as career development and health management (Chan-Olmsted & Wang, 2022). Exposure to such content can enhance listeners’ feelings of competence, self-efficacy, and perceived control over life challenges, thereby reinforcing the belief that useful guidance and resources are accessible when difficulties arise (Turner-McGrievy & Tate, 2013).

Perceived emotional support, by contrast, is largely facilitated through the affective affordances of audio communication. Among the mainstream podcast genres preferred by listeners, hosts frequently employ first-person narration, emotional expressiveness, and self-disclosure, which may foster listeners’ perceived understanding and sense of companionship (Vilceanu, 2025). Beyond the dyadic listener–host relationship, interest-based communities that form around podcast content can further amplify emotional resonance and cognitive identification through shared values, perspectives, and lived experiences, thus strengthening perceived social support (Rohden et al., 2023).

Existing empirical research across mediated communication contexts supports the link between media engagement, perceived social support, and subjective well-being, suggesting that media environments can reinforce individuals’ confidence in the availability of informational and emotional support (Carrotte et al., 2023). By strengthening such confidence, podcast listening may contribute to subjective well-being through enhanced perceived social support. Thus, the following hypothesis is proposed:

**H3.**  *Perceived social support mediates the relationship between podcast listening and subjective well-being among young adults.*

### 2.4. Serial Mediation of Perceived Social Presence and Perceived Social Support

Although H2 and H3 propose distinct mediating pathways linking podcast listening to subjective well-being, reliance on a single mediator is unlikely to fully capture the psychological processes involved. Research in social psychology suggests that immediate experiential states, such as perceived social presence, rarely produce sustained well-being outcomes in isolation (Ostic et al., 2021). Rather, their effects persist when these experiences are internalized into more durable psychological resources, including perceived social support (Yue et al., 2023). In podcast listening, perceived social presence typically represents an immediate experience of perceived companionship. However, due to the unidirectional and asynchronous nature of podcast communication, this experience alone is unlikely to constitute a stable source of support (Euritt, 2022). Its influence on subjective well-being is expected to persist only when it is transformed into a more enduring perception of available support.

The Conservation of Resources theory provides a framework for this transformation. Individuals are motivated to acquire, preserve, and accumulate resources that buffer stress and sustain well-being (Hobfoll et al., 2018). The perceived social presence elicited through podcast listening can be internalized as perceived social support, effectively converting a transient experiential state into stable psychological capital that contributes to subjective well-being (Kim & Wang, 2016).

Social Penetration theory further illuminates this process from a relational perspective. It posits that relational closeness emerges through gradual and sustained self-disclosure (Altman & Taylor, 1973). In podcasts, hosts’ ongoing self-disclosure fosters a stable sense of psychological closeness, which listeners may interpret as indicative of available support (Euritt, 2022; Murphy et al., 2024). Together, these theories suggest a sequential mechanism in which the immediate experiential effects of perceived social presence are internalized as perceived social support, which in turn promotes subjective well-being. Based on this reasoning, the following hypothesis is proposed:

**H4.**  *Perceived social presence and perceived social support sequentially mediate the relationship between podcast listening and subjective well-being among young adults.*

**Figure 1:**
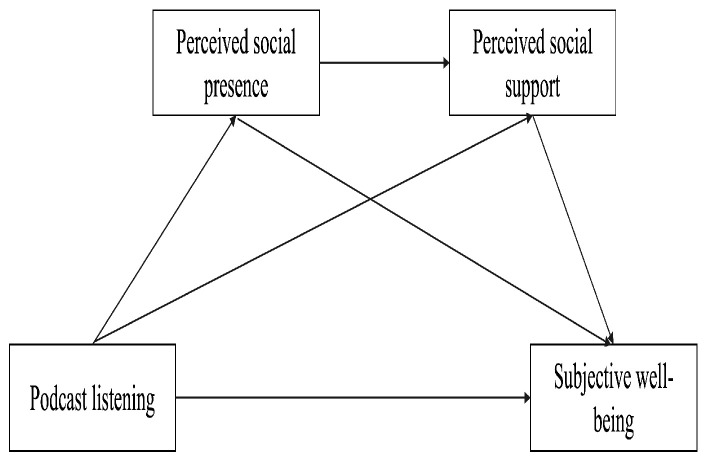
Diagram of the hypothetical serial mediation model.

In summary, this study proposes a serial mediation model, as illustrated in Figure 1. The mediation analysis is intended to examine statistical indirect associations among the variables rather than to provide an empirical test of temporal sequencing or causal effects. Accordingly, the proposed ordering of podcast listening, perceived social presence, perceived social support, and subjective well-being is theoretically derived and should be understood as a hypothesized conceptual framework.

## 3. Materials and Methods

### 3.1. Quantitative Research Process

#### 3.1.1. Participants

In this study, young adults are defined as individuals aged 16–35, consistent with the classification standards of China’s Medium- and Long-Term Young Adults Development Plan (2016–2025) (State Council of China, 2016). Participants were recruited using a convenience sampling strategy. Although this approach may introduce limitations in representativeness when compared with probability-based sampling, it offers practical advantages, including rapid data collection, cost efficiency, and operational flexibility (Etikan et al., 2016).

The analyses focused specifically on young adults with prior podcast listening experience, examining the associations between listening intensity and various dimensions of subjective well-being, rather than the binary effect of podcast use (i.e., whether or not participants listen to podcasts). Therefore, participant recruitment was conducted through major podcast platforms (Xiaoyuzhou and Himalaya) as well as widely used social media platforms (Rednote, Weibo, WeChat, and Douban). Eligibility criteria required that participants: (1) be between 16 and 35 years of age; (2) have listened to at least one podcast episode; and (3) provide podcast listening records or screenshots as verification of listening behavior. To ensure participant privacy and ethical compliance, all submitted screenshots were anonymized, no personal account information was collected, and all verification materials were deleted immediately after confirmation.

Data collection was carried out between 1 June and 30 June 2025. In total, 369 questionnaires were distributed. After removing incomplete or invalid responses, 357 valid questionnaires were retained for analysis, resulting in an effective response rate of 96.75%. All participants were fully informed about the study’s objectives and procedures and provided written informed consent prior to participation. Participation was entirely voluntary, and respondents were explicitly informed of their right to withdraw at any stage without penalty. All data were collected anonymously, used exclusively for academic research purposes, and managed in strict accordance with established ethical and confidentiality standards.

#### 3.1.2. Measures

Self-report measures were used to assess podcast listening, perceived social presence, perceived social support, and subjective well-being. All scales were adapted from validated, internationally recognized instruments via forward and back translation to ensure cross-cultural applicability (Bradley, 1994). Given the specific context of the study, certain items were modified accordingly. Psychometric analyses of the revised scale demonstrated satisfactory reliability and construct validity. Supplementary File S1 shows a comparison of the pre- and post-revision scales in more detail.

Podcast listening scale. Podcast listening was operationalized as the primary independent variable and measured using an adapted version of the Facebook intensity scale (Ellison et al., 2007). The original six-item scale was rated on a 5-point Likert scale (1 = ‘strongly disagree’ to 5 = ‘strongly agree’). To enhance contextual appropriateness for podcast listening, two items reflecting social connectivity (Item 2 ‘I proudly tell people about the podcasts I listen to’ and Item 4 ‘I feel out of touch when I haven’t listened to a podcast for a while’) were removed. This adaptation aimed to better capture core listening intensity and situational dependency, aligning with the study’s focus on foundational media participation rather than social networking behaviors. The modified four-item scale demonstrated acceptable internal consistency, with Cronbach’s α increasing from 0.764 to 0.781 after item deletion. Exploratory factor analysis supported unidimensionality (KMO = 0.741; Bartlett’s test, *p* < 0.001). Confirmatory factor analysis further indicated satisfactory model fit: χ^2^/df = 3.039, GFI = 0.996, AGFI = 0.958, RMSEA = 0.076, CFI = 0.995, TLI = 0.970. These results support the reliability and structural validity of the adapted measure.

Subjective well-being scale. Subjective well-being was assessed using the nine-item scale by Suh and Koo (2011), which covers life satisfaction, positive affect, and negative affect. All items were rated on a 7-point Likert scale (1 = ‘strongly disagree’ to 7 = ‘strongly agree’). The full scale showed good internal consistency (Cronbach’s α = 0.842), with subscale αs of 0.846 (life satisfaction), 0.843 (positive affect), and 0.886 (negative affect). The data were suitable for factor analysis (KMO = 0.811; Bartlett’s test, *p* < 0.001). Confirmatory factor analysis yielded satisfactory fit indices: χ^2^/df = 2.807, GFI = 0.960, AGFI = 0.925, RMSEA = 0.071, CFI = 0.976, TLI = 0.964, supporting the scale’s construct validity in this sample perceived social presence was measured using six items adapted from Lee and Wagner (2002) and Gefen and Straub (2004) on a 5-point Likert scale. The scale, previously validated by Kang and Gretzel (2012) in a park visit context, was adapted for relevance. For example, Item 4 was reworded to: ‘While listening to the podcast, I feel cared for even in the absence of face-to-face interaction with the host.’ The scale indicated acceptable reliability (Cronbach’s α = 0.777). Factorability was good (KMO = 0.833; Bartlett’s test, *p* < 0.001), and CFA supported construct validity: χ^2^/df = 1.69, GFI = 0.986, AGFI = 0.967, RMSEA = 0.044, CFI = 0.988, TLI = 0.979.

Perceived social support was measured using the six-item scale by Y. H. Chen and Keng (2024), with three items each for emotional and informational support (7-point Likert scale). Item 1 (addressing direct comfort from others) was considered more relevant to highly interactive communities and less aligned with this study’s objectives; thus, it was excluded. The remaining five-item scale showed good internal consistency (Cronbach’s α = 0.843), with subscale αs of 0.917 (emotional) and 0.844 (informational). The revised scale demonstrated good factorability (KMO = 0.737; Bartlett’s test, *p* < 0.001). CFA confirmed construct validity: χ^2^/df = 3.00, GFI = 0.987, AGFI = 0.950, RMSEA = 0.075, CFI = 0.992, TLI = 0.980.

The complete questionnaire is available in Supplementary File S2 of the Supplementary Materials.

### 3.2. Qualitative Research Procedure

#### 3.2.1. Qualitative Participants

The qualitative component of this study adopted purposive sampling to select interview participants with theoretically relevant characteristics from among those who had completed the questionnaire survey. Selection was guided by three criteria: (1) participants had successfully completed the questionnaire and met the study’s baseline inclusion requirements; (2) participants represented differing levels of subjective well-being, with individuals drawn from both high and low well-being groups; and (3) sample heterogeneity was enhanced by maintaining a relatively balanced distribution across gender and age.

High and low subjective well-being groups were identified using sample-based quartiles. Participants whose subjective well-being scores fell within the upper quartile were classified as the high well-being group, whereas those in the lower quartile were categorized as the low well-being group. This approach ensured clear differentiation along the study’s central variable. In total, 20 young participants were interviewed, comprising 8 men and 12 women, with a mean age of 24.6 years (SD = 3.87). Detailed participant characteristics are presented in Supplementary File S3.

To examine the influence of podcast listening on young adults and to explore its underlying psychological mechanisms, semi-structured interviews were conducted. The interview protocol encompassed four primary domains: (1) podcast listening practices, including listening motivations and situational contexts; (2) experiences of perceived social presence, focusing on perceived emotional closeness and a sense of interaction with podcast hosts or other listeners; (3) perceived social support, addressing participants’ perceptions of emotional and informational support derived from podcast engagement and its influence on mood and well-being; and (4) subjective well-being, exploring whether and how podcast listening affected participants’ daily lives and emotional states. Interviews continued until theoretical saturation was reached (Charmaz, 2006). The interview guide is provided in Supplementary File S4.

#### 3.2.2. Interview Procedure

All interviews were conducted by the first author, with each session lasting approximately 20–30 min. Depending on participants’ availability and preferences, interviews were carried out either face-to-face or through online text-based communication. To minimize potential discrepancies in data depth across interview modes, a standardized interview guide was used in all sessions, and the interviewer employed active probing and follow-up questions to ensure sufficient detail and clarity. Prior research indicates that when interview topics are clearly specified and the interviewer provides structured guidance, online text-based interviews can generate data of analytical quality comparable to that of face-to-face interviews (Meho, 2006). All interviews were audio-recorded and subsequently transcribed verbatim.

#### 3.2.3. Data Analysis

Qualitative data were analyzed using NVivo 14 software, employing a thematic analysis approach to identify recurring patterns and meanings within participants’ interview narratives. In line with the hypothesis-driven mixed-methods design of this study, the analysis adopted a predominantly deductive orientation, using key constructs from the quantitative phase, perceived social presence, perceived social support, and subjective well-being, as initial analytical categories. At the same time, the analytic process remained open to inductively generated themes that extended beyond the predefined framework, allowing for the identification of unanticipated insights (Braun & Clarke, 2021).

The analysis followed Braun and Clarke’s six-phase thematic analysis framework. First, the first author repeatedly reviewed all interview transcripts to achieve data familiarization. Next, transcripts were systematically coded line by line, with text segments relevant to the research questions identified and labeled. Conceptually related codes were then grouped into preliminary themes, which were iteratively reviewed, refined, and named in accordance with the study’s theoretical orientation.

Multiple strategies were implemented to ensure the trustworthiness of the qualitative findings. An audit trail was maintained across all stages of analysis, documenting coding decisions, analytic memos, and the development of themes. Member checking was conducted with a subset of participants to confirm that the interpretations accurately reflected their lived experiences. In addition, preliminary codes and thematic interpretations were discussed with other researchers to reduce potential bias associated with single-analyst coding and to enhance analytic credibility.

All coding and memo writing were conducted in Chinese to preserve linguistic nuance and contextual meaning. Once thematic saturation was established, representative quotations and thematic summaries were translated into English. To ensure conceptual and semantic equivalence, the translated materials were reviewed through back-translation procedures by a doctoral researcher with expertise in foreign-language translation (Brislin, 1970).

Given that the researcher is also an active podcast listener, reflexivity was consciously integrated throughout the analytic process. Reflexive memos were used to document and monitor the potential influence of the researcher’s positionality on data interpretation. Deliberate efforts were made to distinguish participants’ perspectives from the researcher’s personal experiences, thereby minimizing subjective bias and enhancing analytical transparency.

## 4. Quantitative Research Results

### 4.1. Common Method Bias Test

Because all variables were measured using self-reported data, the potential influence of common method bias cannot be entirely excluded. To mitigate this concern, several procedural remedies were implemented, including the use of reverse-scored items in the questionnaire. As an initial diagnostic, Harman’s single-factor test was conducted, revealing that the first unrotated factor accounted for 29.27% of the total variance, which is below the commonly suggested threshold of 50%. This result suggests that no single factor dominated the covariance among the measures.

CFA was further conducted to assess the potential impact of CMB. The single-factor model demonstrated a poor fit to the data: χ^2^/df = 6.509, GFI = 0.653, AGFI = 0.580, RMSEA = 0.124, CFI = 0.670, TLI = 0.633, whereas the hypothesized four-factor measurement model showed an acceptable fit: χ^2^/df = 2.661, GFI = 0.856, AGFI = 0.822, RMSEA = 0.068, CFI = 0.903, TLI = 0.889. Taken together, these results suggest that common method bias is unlikely to pose a serious threat to the validity of the findings.

### 4.2. Sample Characteristics

As shown in Table 1, the mean age of participants was 23.9 years. Females constituted 73.9% of the sample, aligning with the 2025 China Podcast Industry Report (CICS, 2025), which found 68% of users are female. Most participants were aged between 19 and 26 years (78.2%), and the majority had attained a bachelor’s degree or higher (84%). In terms of marital status, most participants were single (93.3%). In terms of occupational status, students (44.8%) and employed individuals (43.1%) were the largest groups. Income distribution showed that 42.6% of participants earned below RMB 3000 per month, while 29.4% earned between RMB 5001 and 10,000.

With respect to podcast listening behaviors, participants reported an average listening experience of 1.8 years, with ‘1–2 years’ (28.9%) and ‘2–3 years’ (22.4%) being the most common durations. Daily listening time was predominantly between 0.5 and 2 h (65.9%). In terms of podcast genre preferences, participants demonstrated diverse interests. The most frequently preferred categories included self-improvement (69.7%), leisure/entertainment and hobbies (54.3%), music/film/books (49.6%), relationships and emotional life (39.8%), and comedy or talk shows (37.8%).

### 4.3. Group Differences Analysis

Group difference analyses were conducted for demographic characteristics (gender, marital status, education, occupation, income, age) and podcast use patterns (listening duration, daily listening time, genre preference). The results indicate that several demographic and podcast use factors showed significant differences in perceived social presence, perceived social support, and subjective well-being (see Table 2). These analyses describe the distribution of key variables and inform the inclusion of control variables in subsequent models.

### 4.4. Descriptive and Correlation Analysis

Table 3 presents the means, standard deviations, and correlations among the primary variables in this study. Participants reported relatively high mean scores of podcast usage (M = 3.95, SD = 0.75), perceived social presence (M = 4.00, SD = 0.61), perceived social support (M = 5.25, SD = 1.17), and subjective well-being (M = 5.32, SD = 0.88).

Podcast listening was positively correlated with perceived social presence (r = 0.489), perceived social support (r = 0.385), and subjective well-being (r = 0.201), all *p* < 0.01. Perceived social presence was also correlated with perceived social support (r = 0.570), and both were positively associated with subjective well-being (r = 0.344; r = 0.337, *p* < 0.01).

### 4.5. Mediation Analysis

Several demographic variables (gender, age, and education) and podcast-listening characteristics (e.g., listening duration, daily listening time, and podcast genre preference) were significant in bivariate analyses. However, including podcast-listening characteristics in the mediation model did not change the direction, magnitude, or significance of the serial mediation effects. Therefore, the final model controls for gender, age, and education, yielding a parsimonious model of the key mediating relationships.

**Figure 2:**
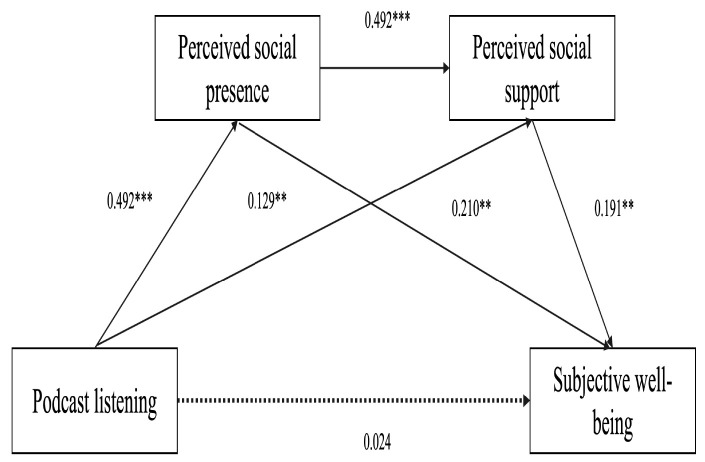
Serial mediation effect model. Note: Non-significant paths are shown in dashed lines, ** *p* < 0.01, *** *p* < 0.001.

Mediation analyses were conducted using the PROCESS macro Model 6 (Hayes, 2017) with 5000 bootstrap resamples to estimate 95% confidence intervals (CIs). As shown in Table 4 and Table 5, the total effect of podcast listening on subjective well-being was significant (β = 0.198, BootSE = 0.052, 95% CI [0.097, 0.300]), supporting H1. Podcast listening was positively associated with perceived social presence (β = 0.492, *p* < 0.001, 95% CI [0.380, 0.610]), which, in turn, was positively associated with subjective well-being (β = 0.210, *p* < 0.01, 95% CI [0.06, 0.36]), consistent with H2. Similarly, podcast listening was positively associated with perceived social support (β = 0.129, *p* < 0.01, 95% CI [0.01, 0.25]), which was further associated with higher subjective well-being (β = 0.191, *p* < 0.01, 95% CI [0.05, 0.35]), supporting H3. Perceived social presence was also positively associated with perceived social support (β = 0.492, *p* < 0.001, 95% CI [0.36, 0.62]), in line with H4. The serial mediation model is shown in Figure 2.

The total indirect effect of podcast listening on subjective well-being via perceived social presence and perceived social support was significant (β = 0.174, BootSE = 0.035, 95% CI [0.114, 0.250]), accounting for 87.7% of the total effect. Specifically, the indirect effect through perceived social presence alone was significant (β = 0.103, BootSE = 0.038, 95% CI [0.033, 0.184]), representing 52.0% of the total effect. The indirect effect through perceived social support alone was β = 0.025 (BootSE = 0.017, 95% CI [0.001, 0.068]), accounting for 12.4%.

The serial mediation pathway was also significant (β = 0.046, BootSE = 0.019, 95% CI [0.013, 0.087]), contributing 23.3% of the total effect. In contrast, the direct effect of podcast listening on subjective well-being was not significant (β = 0.024, BootSE = 0.057, 95% CI [−0.088, 0.137]), indicating that the relationship between podcast listening and subjective well-being is primarily explained by the mediating roles of perceived social presence and perceived social support. Perceived social presence appears to play the strongest mediating role, whereas perceived social support plays a supplementary mediating role in the observed associations.

### 4.6. Robustness Analysis

To assess the robustness of the serial mediation model, two analyses were conducted. First, using unstandardized scores while controlling for gender, age, and education, podcast listening positively predicted perceived social presence (B = 0.405, *p* < 0.001), which predicted perceived social support (B = 0.936, *p* < 0.001). Both mediators predicted subjective well-being (B = 0.300 and 0.144, *p*s ≤ 0.002), and the direct effect was non-significant (B = 0.029, *p* = 0.671), indicating a full mediation pattern in the statistical model. Second, controlling additionally for podcast-listening characteristics, the serial indirect pathway remained significant (indirect effect = 0.042, 95% CI [0.004, 0.091]), and the total indirect effect was robust (0.185, 95% CI [0.113, 0.276]). The direct effect of podcast listening on perceived social support became non-significant, indicating that use patterns explain some superficial association, but the central mediating role of perceived social presence remains intact.

In addition, an alternative sequence model (Model 1: PL → PSS → PSP → SWB) and a reverse-order model (Model 2: SWB → PSP → PSS → PL) were tested. Total indirect effects were significant in both models (Model 1: β = 0.205, 95% CI [0.131, 0.296]; Model 2: β = 0.154, 95% CI [0.100, 0.219]). The originally hypothesized model accounted for a larger proportion of the indirect effect (23.3%) than the alternatives (17.5% and 12.2%), suggesting greater consistency with the observed data. Significant effects in alternative specifications indicate that the associations are not strictly unidirectional; these results reflect robust statistical associations rather than definitive causal ordering, although theoretically, positioning podcast listening as the predictor aligns with the study framework. Overall, the statistical mediation patterns are robust to demographics and listening patterns (see Supplementary File S5).

## 5. Qualitative Research Results

Analysis of the data identified four main themes, each encompassing multiple subthemes, which capture young adults’ perceptions of podcast listening and its impact on subjective well-being. Detailed coding specifications are provided in Supplementary File S6.

### 5.1. Experiencing Perceived Social Presence in Podcast Listening

First, perceived social presence emerges from the role of podcasts as ambient background audio embedded in everyday life. Participants frequently described podcasts as a form of stable companionship that accompanies solitary activities such as commuting, performing household chores, or studying. This continuous auditory presence was described by participants as enabling them to feel accompanied without disrupting their ongoing activity. As P05 noted:

*I usually listen to podcasts during my commute, while doing my makeup, or whenever I’m feeling a bit down.*

Participants further emphasized that podcasts gradually become embedded within their daily temporal rhythms. Listening in the early morning or before bedtime was frequently described as a habitual practice and a stable temporal anchor in everyday life. As P08 explained:

*I usually turn to podcasts proactively when I find myself tossing and turning before bed; they have become my preferred way to unwind and fall asleep.*

This self-regulated listening rhythm afforded listeners a sense of temporal control in their daily lives. Particularly during periods of emotional distress or heightened stress, participants perceived the predictability and continuity of podcast listening as facilitating emotional regulation and helping to restore a sense of psychological order. As P02 stated:

*When I’m not feeling great and need input beyond music, I turn to podcasts.*

Second, perceived social presence was also constructed through auditory cues and immersive listening experiences. Participants frequently described moments of deep psychological absorption during podcast listening, with some likening the experience to participating in an ongoing conversation. For instance, P01 remarked:

*Yang Tianzhen’s podcast repeatedly made me feel the host’s ‘exceptional presence,’ as if she were chatting face-to-face with me.*

Across interviews, participants highlighted sensitivity to vocal details (intonation, pitch, pauses, rhythm), shaping perceived social presence. P01 elaborated on this auditory experience as follows:

*When I feel immersed in a podcast host’s delivery, it hinges on their voice, tone, and the sincerity of their content. A soft, rich voice naturally conveys warmth and approachability, instantly closing the distance.*

Compared with the more formal, authoritative, or projection-oriented vocal styles typically associated with traditional radio broadcasting, podcast voices were often characterized as softer, calmer, and more intimate. This vocal quality was reported by participants to narrow the perceived psychological distance between hosts and listeners. P05 illustrated this point by referring to a specific episode:

*Siwen’s podcast featured an episode titled ‘My Grandma Was More Bitter Than Coffee and More Stout Than Kunlun.’ When Siwen introduced her grandmother to listeners, she vividly recounted her Sichuan dialect and personality, painting a lively portrait of an elderly woman.*

Importantly, participants did not attribute these vocal qualities to the host’s biological sex. Instead, they understood them as an expressive and communicative style. Several participants noted that regardless of gender, hosts who conveyed emotional involvement and sincerity through their voices were more likely to evoke a strong sense of perceived social presence.

Third, perceived social presence was further reinforced through parasocial interactions between listeners and podcast hosts. Although listeners remained physically silent, many participants reported spontaneous cognitive and emotional responses during listening, which drew them into the unfolding discussion and intensified their sense of presence. P05 vividly described this experience:

*I often find myself responding to the host’s opinions in my head. When they talk about something funny, I feel happy along with them; when they share something sad, I might even tear up.*

Some participants conceptualized this interaction as an internal psychological dialog. While listening, they resonated emotionally with the host’s narratives and mentally formulated responses, whether in agreement or critique. Although these reactions did not manifest as overt speech, they constituted a one-way yet highly engaged interactive experience. As P08 explained:

*This subtle sense of interaction is fundamentally determined by the content itself. Vividly engaging content that captures attention draws listeners unconsciously into the emotional flow.*

### 5.2. Perceived Social Support in Podcast Listening

At the emotional level, podcasts provided listeners with a sense of being acknowledged and understood. Multiple interviewees noted that programs addressing social issues commonly experienced by young adults, such as struggles related to personal growth, family-of-origin dynamics, or identity exploration, evoked a strong feeling of being understood without requiring explicit self-disclosure. As P12 recalled:

*Listening to the ‘Pleasing Personality’ episode of the Aotu podcast, when the host said, ‘I’m always afraid to refuse others, then regret it afterward,’ it instantly resonated with me. In that moment, I felt, ‘Finally, someone gets me’.*

Therefore, for some young listeners who experienced limited emotional support within their offline social networks, podcasts were perceived by some participants as an important supplementary source of emotional reassurance. P02 described their sustained engagement with a particular program:

*My favorite remains After School. They truly gave me courage. Life may hold countless troubles, but as long as you’re willing to set out, the world holds endless beauty waiting to be explored.*

In addition, by openly sharing personal struggles, failures, or transitions, hosts rendered emotional vulnerability legitimate and relatable. P13, who experienced a period of personal difficulty, explained:

*Last year, when I was unemployed, staying home all day made me incredibly anxious. I listened to podcasts when I couldn’t sleep at night, especially hosts sharing stories about career transitions and the pitfalls of entrepreneurship. It made me realize I wasn’t alone in facing a low point. Back then, podcasts were like a pillar of support, really important.*

Podcasts also provided informational support. This form of perceived informational support did not rely on direct interpersonal exchange but instead emerged from exposure to others’ accumulated knowledge, reflections, and lived experiences. P14 explained their motivation for selecting podcast content:

*The podcasts I choose offer fresh perspectives. For example, they might explore public transportation systems or medical topics, areas far beyond my daily knowledge. This often broadens my horizons.*

Participants particularly highlighted the role of podcasts in systematically unpacking real-world challenges, which helped them reframe their own situations. On topics such as workplace relationships, career development, and interpersonal communication, podcast content was frequently delivered through case analyses, Q&A segments, or experience-based summaries. For instance, P01 noted:

*Yang Tianzhen’s podcast had a dedicated Q&A episode on workplace communication and career planning, offering highly specific and directly applicable methods. After listening, I felt my thinking opened up, and I later tried some of those techniques at work.*

Similar accounts appeared across multiple interviews. Importantly, participants did not perceive such guidance as prescriptive or authoritative. Rather, it was understood as a flexible cognitive framework. Participants described that this informational support enhanced their perceived sense of competence and efficacy in navigating uncertainty.

Podcast-related communities also emerged as a meaningful source of perceived social support. Several participants noted that simply being aware of a shared listener base fostered a sense of belonging to an imagined collective. Even in the absence of direct interaction, this perceived co-presence was experienced as supportive. As P11 stated:

*We also have a listener community where people discuss the show and actively contribute ideas.*

This suggests that listeners derive a sense of community through imagined collective participation during the listening process. Moreover, listeners occasionally gather offline—further reinforcing this sense of belonging. P11 elaborated:

*We even organized an offline gathering for the ninth anniversary of Aotu Radio, and we’ve become real-life friends.*

### 5.3. Sequential Experiences of Podcast Listening

Participants’ narratives indicate that the sense of perceived social presence derived from podcast listening is not limited to the immediate listening episode. Rather, listeners commonly described a temporally unfolding psychological process. When podcast listening becomes routinized within everyday life, the sense of presence generated during listening stabilizes and accumulates over time. Through repeated exposure to the same programs, hosts’ voices become increasingly familiar and predictable. As a result, participants reported that the auditory presence of the host was no longer experienced as intermittent or unfamiliar, but rather as continuous, reliable, and emotionally accessible. P04 described this transition as follows:

*The hosts of the two podcasts I regularly listen to share glimpses of their lives, and hearing them so often makes it feel like they’re close friends.*

In particular, when podcast content resonates with listeners’ current emotional states or lived circumstances, participants frequently reported an intensified sense of being understood. Although the content was not directed at them personally, listeners interpreted the narratives and viewpoints as highly applicable to their own situations. This perceived relevance was often described by participants as facilitating emotional relief and cognitive reappraisal. P06 provided a detailed account of this process:

*I was feeling really frustrated about some academic issues, and after listening to one podcast episode, I realized I’d been trapped by a kind of perfectionism that’s followed me since childhood. Listening to the podcast eased my stress and helped me adjust my mindset. Hearing others share their experiences made me realize I wasn’t alone in facing similar challenges. Their stories told me, ‘Don’t be anxious, maybe just put in a little more effort’.*

Across interviews, similar accounts revealed that emotional relief was frequently attributed not only to the informational content of the podcast but also to the sustained sense of companionship embedded within the listening experience. Participants consistently linked moments of psychological relief to the perception of sharing experiences with others over time, rather than to isolated listening episodes.

### 5.4. Additional Experiential Patterns Identified in Interviews

Beyond the mechanisms identified through quantitative modeling, the interview data revealed several experiential dimensions that are not fully captured by standardized survey measures. Most notably, some participants reported a pronounced sense of perceived social presence, without a corresponding perception of emotional or instrumental support. For these listeners, podcasts functioned primarily as an ambient presence or affective backdrop, rather than as an explicit source of reassurance, guidance, or encouragement. P14 vividly described this form of perceived social presence:

*Actually, the podcasts I enjoy tend to have hosts who create this sense of companionship, you find yourself drawn into a conversation with the host. You feel like you’re right there in the moment, almost as if you’re watching them have a conversation.*

However, when reflecting on perceived social support, the same participant articulated a markedly different interpretation of the listening experience:

*Since I don’t seek emotional comfort or practical advice from podcasts, what he offers me is a different perspective on the world, not the kind of emotional support I’d turn to or solutions to specific problems.*

This pattern reflects a ‘high perceived social presence–low perceived social support’ experience. These accounts suggest a potential boundary condition for the serial mediation model examined in the quantitative analysis. Specifically, perceived social presence alone may be insufficient to generate perceptions of social support, as reflected in some participants’ accounts. Instead, the transformation of experiential presence into perceived support appears contingent on listeners’ interpretive frames, including their motivations for listening, expectations of media use, and the perceived relevance of podcast content to their personal needs or life circumstances.

## 6. Discussion

### 6.1. Podcast Listening Among Young Adults and Subjective Well-Being

This study provides empirical support for Hypothesis H1, indicating a positive association between podcast listening and subjective well-being among young adults. Podcasts offer opportunities for knowledge acquisition, exposure to diverse viewpoints, and the development of pseudo-social relationships with hosts. Through repeated and sustained engagement, listeners may gradually perceive themselves as accumulating experiential and cognitive resources (Ryan & Deci, 2000). Over time, these resources may become incorporated into daily routines and be experienced as habitual tools for emotional regulation. These resources may be perceived as helping individuals cope with daily stressors, adopt more adaptive cognitive appraisals, and experience positive emotions—factors that are consistently linked to higher levels of subjective well-being (Diener et al., 1999).

Moreover, frequent podcast listening often develops into a ritualized habit embedded within daily routines, such as commuting, leisure breaks, or study intervals. When incorporated into these predictable temporal contexts, podcast listening may serve an emotion-regulation function. Individual listening episodes are commonly associated with anticipated experiences of enjoyment, relaxation, or motivational uplift (McKinney & Page, 2009). Over time, repeated exposure to these predictable positive affective states may be associated with a more favorable emotional baseline, characterized by increased positive affect and reduced negative affect. Consistent with the previous findings (J. L. Li et al., 2025), podcasts may function as habitual emotional regulation tools. Importantly, the association between podcast listening and subjective well-being appears to be contingent on listening frequency and sustained engagement over time.

### 6.2. How Perceived Social Presence Mediates the Relationship Between Podcast Listening and Subjective Well-Being 

This study provides empirical support for Hypothesis H2, suggesting that perceived social presence operates as a mediating mechanism in the association between podcast listening and subjective well-being among young adults. Consistent with prior research, young adults who perceive richer communicative cues through podcasts tend to experience a stronger sense of others’ psychological presence (Kang & Gretzel, 2012), which is in turn associated with more positive psychological outcomes. Although Short et al. (1976) originally argued that audio-based media convey weaker perceived social presence than face-to-face communication, contemporary podcasting practices have substantially expanded the social affordances of audio media. Through conversational formats, sustained host–listener relationships, and personalized delivery styles, podcasts have enhanced interactivity and humanized mediated communication, positioning voice as a central conduit for perceived social presence.

The appeal of podcasts among young adults appears to lie primarily in the communicative power of the human voice. Vocal cues, including intonation, emotional expressiveness, pacing, and rhythm, effectively transmit affective and relational information, often surpassing text-based or purely visual stimuli in evoking immersion and perceived co-presence (Perks et al., 2019). This heightened sense of perceived social presence may strengthen listeners’ psychological engagement by fostering feelings of involvement, immediacy, and emotional attachment (Nass & Brave, 2005). For many young listeners, the experience of being accompanied by a human voice may outweigh the instrumental value of the content itself. Media psychology research further suggests that such immersive presence experiences constitute an important pathway through which media use promotes positive affective states and subjective well-being (Dowling & Miller, 2019).

Moreover, the intimate soundscape produced through headphone-based listening enables podcasts to simulate one-on-one or near face-to-face interaction across diverse everyday contexts (Swiatek, 2018). This form of immersive perceived social presence may be experienced as emotionally comforting and validating, thereby supplying psychological resources that are associated with enhanced subjective well-being (Kang & Gretzel, 2012). Nevertheless, as indicated by the qualitative findings, perceived social presence alone does not uniformly translate into improved well-being. Its beneficial effects appear to depend on whether listeners cognitively and emotionally interpret this presence as meaningful, supportive, or relevant to their own lived experiences.

### 6.3. How Perceived Social Support Mediates the Relationship Between Podcast Listening and Subjective Well-Being

The findings provide empirical support for Hypothesis H3, suggesting that perceived social support serves as a mediating factor in the association between podcast listening and subjective well-being, in line with existing theoretical and empirical evidence (Eagle et al., 2018). Perceived social support reflects individuals’ subjective appraisals of the availability, adequacy, and reliability of supportive resources, underscoring that media-provided support must be cognitively recognized and internalized to be experienced as psychologically meaningful (Cobb, 1976).

Podcast listening may enhance perceived social support through both informational and emotional pathways. Through the dissemination of knowledge, experiential narratives, and practical advice, podcasts may contribute to listeners’ perceived sense of cognitive control and problem-solving capacity (Zhang et al., 2023). This informational support may reduce feelings of uncertainty and isolation, thereby providing psychological resources that individuals may draw upon when managing life challenges. Simultaneously, hosts’ empathetic storytelling, authentic self-disclosure, and sustained engagement with specific audiences can foster emotional resonance that transcends physical and spatial boundaries. Such affective connections enable listeners to feel understood, cared for, and emotionally affirmed, reinforcing perceptions of supportive availability (Edwards et al., 2021). This mediating mechanism is consistent with Eagle et al.’s observation that perceived social support exerts a more stable and enduring influence on subjective well-being than objectively enacted support behaviors (Eagle et al., 2018). Through repeated exposure to supportive content and emotional cues, young listeners may thus construct a subjective sense of a dependable support network that may function as a psychological buffer.

Notably, however, the mediating effect of perceived social support accounts for only approximately 12.4% of the total effect. This relatively modest proportion may be attributable to the unidirectional and asynchronous nature of podcast communication. Unlike social media platforms that enable reciprocal interaction and personalized feedback, podcast listeners rarely receive direct responses from hosts. This structural limitation may constrain the extent to which listeners develop stable beliefs regarding the availability of individualized support, thereby attenuating the overall mediating influence of perceived social support.

### 6.4. Serial Mediation from Perceived Social Presence to Perceived Social Support

This study provides empirical support for Hypothesis H4, suggesting a serial mediation pathway linking podcast listening, perceived social presence, perceived social support, and subjective well-being. Specifically, experiences of perceived social presence generated during podcast listening appear to involve active cognitive and emotional processing before being internalized as perceived social support, which is in turn associated with subjective well-being.

This pattern aligns with the relationship-deepening logic articulated in Social Penetration Theory (Altman & Taylor, 1973). Although podcast communication is unidirectional, relational continuity is sustained through listeners’ repeated engagement over time. As a result, the host–listener relationship is cognitively redefined, moving from episodic content consumption toward a predictable, quasi-relational bond characterized by emotional familiarity and reduced relational uncertainty. Such stability provides a psychological foundation upon which perceived social presence can be reinterpreted as a potential source of support.

The transformative mechanism further involves a shift from content resonance to supportive meaning construction. When podcast narratives align with listeners’ lived experiences or current challenges, listeners may personalize the host’s reflections and interpret them as indirectly relevant guidance. This dynamic is consistent with COR theory (Hobfoll et al., 2018), which posits that individuals actively convert positive environmental cues into enduring psychological resources. In this way, situational experiences of perceived social presence may be gradually interpreted as resource-oriented perceptions of social support, thereby outlining a plausible sequential pathway associated with enhanced subjective well-being.

The qualitative findings also reveal a ‘high perceived social presence–low perceived social support’ pattern, indicating that strong experiential presence does not automatically translate into perceived support. This observation highlights a critical distinction in media effects research between immediate, immersive psychological experiences and sustained, resource-based beliefs. The transition from presence to support appears to depend on several moderating conditions, including: (1) the depth and relevance of podcast content to listeners’ personal concerns; (2) listeners’ cognitive orientations and interpretive tendencies toward media experiences; (3) listening contexts characterized by fragmentation or multitasking, which may weaken emotional engagement and hinder the internalization of supportive meanings. Together, these factors help explain why perceived social presence alone may be insufficient to generate perceived social support and underscore the conditional nature of the proposed serial mediation pathway.

Building on the empirical findings from Hypotheses H1–H4, the mechanisms identified in this study—linking podcast listening, perceived social presence, perceived social support, and subjective well-being—can be situated within broader discussions of audio media use and socio-cultural conditions. While the present study focuses specifically on podcasts, similar mechanisms may also be relevant to other forms of audio-based media, such as in-car audio systems, audiobooks, and digital audio communities. Despite differences in format or content, the companion-like qualities of these media and the human voice may evoke comparable subjective perceptions that are theoretically relevant to listeners’ well-being (Menon, 2025).

Moreover, the manifestation of these mechanisms among Chinese young adults is shaped by their particular socio-cultural context. This population faces a highly competitive environment and often experiences loneliness amid rapid urbanization (Zhan et al., 2021; M. Li, 2021). However, such stress-related experiences are not unique to China: research indicates that roughly one-fifth of young people globally encounter varying degrees of mental health challenges (T. Chen et al., 2024). Conceptualizing mental health as a continuum encompassing stress, adaptation, and resilience (WHO, 2022), young adults’ use of media to cope with academic pressure, identity anxiety, future uncertainty, and family expectations reflects a phenomenon with notable cross-cultural commonality (Wright et al., 2025). Accordingly, the pathway to well-being via podcasts observed in this study may represent a potential coping mechanism for recurring psychological challenges within global digital youth culture. However, its effectiveness is likely influenced by cultural and institutional contexts.

## 7. Conclusions

### 7.1. Main Conclusions

This study yields four principal conclusions. First, podcast listening is positively associated with subjective well-being among young adults. Second, perceived social presence emerges as a statistically significant mediator in the association between podcast listening and subjective well-being. Third, perceived social support shows a modest yet statistically meaningful mediating effect within this association. Fourth, perceived social presence and perceived social support form a statistically supported sequential mediation pattern in the proposed model. Together, they delineate a theoretically informed pathway through which podcast listening is associated with subjective well-being.

### 7.2. Theoretical Implications

This study advances understanding of the sequential pattern through which media engagement is associated with subjective well-being. By testing a serial mediation model, the findings suggest that media engagement is indirectly associated with subjective well-being through a sequential pathway. Within this pathway, perceived social presence is progressively reinterpreted as a more enduring outcome via perceived social support, highlighting a process-oriented perspective beyond simple direct-effect models. The study also shows that perceived social presence can emerge in purely audio-based, non-interactive media environments. Sustained auditory engagement allows it to be reconceptualized as an experiential psychological state rather than an interaction-dependent construct. In this process, perceived social support can develop even without explicit interpersonal communication. Perceived social presence may function as a psychological bridge, whereby transient feelings of companionship are associated with more stable support perceptions and highlight users’ active role in meaning-making and psychosocial resource accumulation. Focusing on Chinese young adults, the findings also illustrate how sociocultural pressures shape media use motivations and well-being outcomes, demonstrating that low-demand auditory media may function as accessible emotional resources in everyday life. Collectively, these contributions challenge the assumption that higher interactivity necessarily enhances well-being and offer theoretically grounded insights into young adults’ digital mental health.

### 7.3. Limitations and Future Research Directions

Several limitations of this study should be acknowledged. First, the cross-sectional and self-report design precludes definitive causal inferences regarding the proposed sequential relationships among podcast listening, perceived social presence, perceived social support, and subjective well-being. While the serial mediation model is theoretically grounded and supported by statistical associations, the temporal ordering of variables cannot be empirically verified, and the observed indirect effects should be interpreted as statistical associations rather than evidence of causal mediation. The fact that alternative model specifications also yielded significant indirect effects further underscores the limitations of cross-sectional mediation analyses in establishing causal direction. Future research employing longitudinal, experimental, or experience-sampling designs is needed to examine the dynamic evolution of podcast engagement and its cumulative effects on subjective well-being.

Second, the sample is skewed toward younger, predominantly female participants who are relatively highly engaged podcast users, and it does not include non-listeners. Consequently, the findings are limited to associations between listening intensity and subjective well-being among existing listeners, and cannot be generalized to the broader youth population or interpreted as evidence of the effects of starting to listen to podcasts. Subsequent studies should include more heterogeneous and nationally representative samples across age groups, occupations, socioeconomic strata, and cultural contexts, in order to assess the robustness and external validity of the proposed mechanisms.

Third, the study focused primarily on intimacy-oriented and emotionally resonant podcast genres, which dominate both the quantitative sample and the podcasts most frequently cited in the qualitative interviews. As a result, the findings provide limited insight into more information-driven genres, such as technology or sports podcasts, where the relevant psychological mechanisms may relate more to information acquisition, cognitive engagement, or instrumental use rather than emotional companionship. This focus reflects the broader tendency toward emotionally oriented content in the contemporary Chinese podcast ecosystem, but it also constrains the generalizability of the results to other podcast types. Future research could systematically examine how podcast genres, narrative styles, and perceived production quality moderate the proposed mechanisms.

Finally, this study did not account for potentially influential latent variables, such as personality traits, baseline mental health, or media-use motivations, and the measurement instruments for perceived social presence and perceived social support were adapted specifically to the podcast context. Although this contextualization enhances ecological validity, it may have excluded broader theoretical dimensions of these constructs. Future research could employ more comprehensive measurement approaches and advanced structural modeling techniques to explore multi-level, multi-factor mechanisms linking podcast engagement and subjective well-being.

## Figures and Tables

**Table 1:** Demographic characteristics and podcast listening habits of participants.

Variable Category	Item	Frequency (n)	Percentage (%)
Gender	Male	93	26.1
	Female	264	73.9
Age	16–18	11	3.1
	19–22	127	35.6
	23–26	152	42.6
	27–30	40	11.2
	31–35	27	7.6
Education level	High school/Technical secondary school or below	16	4.5
	Junior college	41	11.5
	Bachelor’s degree	240	67.2
	Master’s degree or above	60	16.8
Marital status	Single	333	93.3
	Married	24	6.7
Occupation	Student	160	44.8
	Employed	154	43.1
	Freelancer	35	9.8
	Full-time homemaker	2	0.6
	Other	6	1.7
Income level	Below RMB 3000	152	42.6
	RMB 3001–5000	77	21.6
	RMB 5001–10,000	105	29.4
	Above RMB 10,000	23	6.4
Listening duration	Less than 6 months	40	11.2
	6 months–1 year	72	20.2
	1–2 years	103	28.9
	2–3 years	80	22.4
	3–4 years	37	10.4
	More than 4 years	25	7.0
Daily listening time	Less than 0.5 h	43	12.0
	0.5–1 h	128	35.9
	1–2 h	107	30.0
	2–4 h	64	17.9
	More than 4 h	15	4.2
Preferred podcast genres	Self-improvement	249	69.7
	Leisure/Entertainment and Hobbies	194	54.3
	Music, Film and Books	177	49.6
	Relationships and Emotional Life	142	39.8
	Comedy and Talk Shows	135	37.8
	Society, Culture and History	129	36.1
	Mystery/Thriller	126	35.3
	Fitness and Health	77	21.6
	News	75	21.0
	Business	72	20.2
	Arts	62	17.4
	Fashion and Beauty	59	16.5
	Technology	49	13.7
	Parenting and Family	35	9.8
	Sports	16	4.5
	Religion	14	3.9

Percentages are based on the valid sample (N = 357). Multiple selections were allowed for ‘Preferred podcast genres,’ so the total percentages exceed 100%. A more detailed classification of podcast types is provided in Supplementary File S7.

**Table 2:** Group differences in perceived social presence, perceived social support, and subjective well-being.

Variable Category	Grouping Variable	Podcast Listening	Perceived Social Presence	Perceived Social Support	Subjective Well-Being
Gender (t)	Male vs. Female	1.754	0.893	4.053 ***	2.112 *
Marital status (t)	Single vs. Married	−0.753	0.061	−0.200	−1.305
Age group (F)	16–18/19–22/23–26/27–30/31–35	1.133	4.561 ***	2.179	1.592
Education level (F)	High school (Technical secondary school) or below/Junior college/Bachlor’s degree/Master’s degree or above	0.922	5.113 **	5.395 ***	0.780
Occupation (F)	Student/Employed/Freelance/Homemaker/Other	0.546	0.912	0.727	1.680
Income level (F)	≤3000/3001–5000/5001–10,000/≥10,000	0.957	4.741 **	2.182	0.164
Listening duration (years) (F)	<6 months/6 months–1 year /1–2 years /2–3 years/3–4 years/≥4 years	6.092 ***	3.969 **	3.139 *	2.633 *
Daily listening time (F)	<0.5 h/0.5–1 h/1–2 h/2–4 h/>4 h	17.095 ***	2.674 *	5.090 ***	2.956 *
Podcast genre preference (F)	18 podcast genre categories	6.804 *	10.244 *	33.734 *	2.201 *

Values represent t-statistics for binary variables and F-statistics for multi-group comparisons. * *p* < 0.05, ** *p* < 0.01, *** *p* < 0.001.

**Table 3:** Descriptive statistics and Pearson correlations of key variables among participants.

Variable	M	SD	1	2	3	4
1. Podcast Listening	3.95	0.75	1			
2. Perceived social presence	4.00	0.61	0.489 **	1		
3. Perceived Social Support	5.25	1.17	0.385 **	0.570 **	1	
4. Subjective Well-Being	5.32	0.88	0.201 **	0.344 **	0.337 **	1

N = 357. Values above the diagonal represent Pearson correlation coefficients. ** *p* < 0.01.

**Table 4:** Regression analysis.

Predictor	Perceived Social Presence			Perceived Social Support			Subjective Well-Being		
	β	SE	t	β	SE	t	β	SE	t
Gender	−0.012	0.046	−0.25	−0.160	0.043	−3.73 ***	−0.075	0.051	−1.48
Education	0.071	0.049	1.45	−0.026	0.045	−0.57	0.106	0.052	2.03 *
Age	−0.164	0.048	−3.40 ***	−0.053	0.045	−1.16	−0.080	0.053	−1.53
Podcast Listening	0.492	0.046	10.63 ***	0.129	0.049	2.63 **	0.024	0.057	0.43
Perceived Social Presence	—	—	—	0.492	0.049	9.97 ***	0.210	0.065	3.25 **
Perceived Social Support	—	—	—	—	—	—	0.191	0.062	3.10 **
Model Fit	R = 0.513	R^2^ = 0.263	F(4, 352) = 31.46 ***	R = 0.609	R^2^ = 0.371	F(5, 351) = 41.38 ***	R = 0.404	R^2^ = 0.164	F(5, 351) = 11.41 ***

* *p* < 0.05, ** *p* < 0.01, *** *p* < 0.001. Perceived Social Presence and Perceived Social Support were entered sequentially in the mediation models.

**Table 5:** Total, direct, and indirect effects of podcast listening on subjective well-being.

Effect Type	Path	Standardized Effect (βcs)	BootSE	BootLLCI	BootULCI	% of Total Effect
Total Effect	PL → SWB	0.198	0.052	0.097	0.300	-
Direct Effect	PL → SWB (c′)	0.024	0.057	−0.088	0.137	-
Indirect Effect	PL → PSP → SWB	0.103	0.038	0.033	0.184	52.0%
Indirect Effect	PL → PSS → SWB	0.025	0.017	0.001	0.068	12.4%
Chain Indirect Effect	PL → PSP → PSS → SWB	0.046	0.019	0.013	0.087	23.3%
Total Indirect Effect	-	0.174	0.035	0.114	0.250	87.7%

PL = Podcast Listening; PSP = Perceived Social Presence; PSS = Perceived Social Support; SWB = Subjective Well-Being.

## Data Availability

The data that support the findings of this study are available from the author upon reasonable request.

## Supplementary Materials

The following supporting information can be downloaded at: https://www.mdpi.com/article/10.3390/bs16020267/s1.
