# Supplementary material for: Podcast Listening, Perceived Social Presence, Perceived Social Support, and Subjective Well-Being Among Chinese Young Adults: Sequential Explanatory Mixed Methods Study
Source: Behav Sci (Basel). 2026 Feb 11;16(2):267. doi: 10.3390/bs16020267 (PMC12938595; doi:10.3390/bs16020267)
Supplement: Supplementary file 1 [file behavsci-16-00267-s001.zip › Supplementary File S7.pdf]

Supplementary File S7. Refined classification of podcast genres and subcategories

Broad Category	Refined Subcategories / Examples
Self-improvement	Career development; Personal growth and life philosophy; Study skills and exam preparation; Financial literacy and self-management; Mental well-being and emotional regulation
Leisure / Entertainment and Hobbies (Casual and lighthearted storytelling)	Daily life sharing and casual talk; Gaming and e-sports; Travel and outdoor activities; Food, cooking, and lifestyle hobbies; Handcrafts, photography, and creative hobbies
Music, Film and Books	Music appreciation and playlists; Film and television reviews; Book discussions and reading clubs; Cultural criticism and recommendation podcasts
Relationships and Emotional Life	Romantic relationships and dating; Friendship and peer relationships; Family and intergenerational relations; Emotional sharing and personal storytelling
Comedy and Talk Shows	Stand-up comedy and humorous monologues; Conversational talk shows; Satirical commentary and parody
Society, Culture and History	Social issues and public discussion; Cultural observation and commentary; History storytelling and popular historiography
Mystery / Thriller	True crime and real cases; Fictional suspense and thrillers; Paranormal and urban legends
Fitness and Health	Physical fitness and exercise guidance; Health knowledge and wellness advice; Psychological health and stress management
News	Current affairs and political news; Social news and commentary; Industry and niche news updates
Business	Entrepreneurship and startups; Workplace management and leadership; Economic trends and market analysis
Arts	Visual arts and design; Performing arts (theatre, dance); Art criticism and aesthetics
Fashion and Beauty	Fashion trends and styling; Beauty, skincare, and cosmetics; Body image and self-presentation
Technology	Consumer technology and digital life; Artificial intelligence and innovation; Science communication and popular science
Parenting and Family	Parenting experience sharing; Family education and child development; Work–family balance
Sports	Sports news and commentary; Athlete stories and sports culture; Fitness-related sports discussions
Religion	Religious teachings and belief discussion; Spiritual reflection and moral philosophy
