# Supplementary material for: Podcast Listening, Perceived Social Presence, Perceived Social Support, and Subjective Well-Being Among Chinese Young Adults: Sequential Explanatory Mixed Methods Study
Source: Behav Sci (Basel). 2026 Feb 11;16(2):267. doi: 10.3390/bs16020267 (PMC12938595; doi:10.3390/bs16020267)
Supplement: Supplementary file 1 [file behavsci-16-00267-s001.zip › Supplementary File S1.pdf]

Supplementary File S1: Comparison of Pre- and Post-Scale

<table>
<tr><th>Scale</th><th>Original Item</th><th>Modified Item</th><th>Reason for Modification</th><th>Item Retained/Removed</th><th>Psychometric Notes (Initial Item Pool)</th><th>Psychometric Notes (Final Scale)</th></tr>
<tr><td rowspan="6">Podcast Listening Intensity (Ellison et al., 2007)</td><td>1. Facebook is part of my everyday activity.</td><td>1.Podcast is part of my everyday activity.</td><td>Adjusted wording for context</td><td>Retained</td><td rowspan="6">Cronbach's α = .764; EFA KMO = .795; Bartlett's test p < .001;CFA: χ^2/df = 5.367, GFI = .953, AGFI = .889, RMSEA = .111, CFI = .926, TLI = .877</td><td rowspan="6">Cronbach's α = .781; EFA KMO = .741; Bartlett's test p < .001;CFA: χ^2/df = 3.039, GFI = .996, AGFI = .958, RMSEA = .076, CFI = .995, TLI = .970</td></tr>
<tr><td>2. I am proud to tell people I'm on Facebook.</td><td>—</td><td>Removed because podcast listening is typically individual and asynchronous.</td><td>Removed</td></tr>
<tr><td>3. Facebook has become part of my daily routine.</td><td>3. Podcast has become part of my daily routine</td><td>Adjusted wording for context</td><td>Retained</td></tr>
<tr><td>4. I feel out of touch when I haven't logged onto Facebook for a while.</td><td>—</td><td>Removed to reduce social connectivity bias.</td><td>Removed</td></tr>
<tr><td>5. I feel I am part of the Facebook community.</td><td>5. I feel I am part of the podcast community.</td><td>Adjusted wording for context</td><td>Retained</td></tr>
<tr><td>6. I would be sorry if Facebook shut down.</td><td>6. I would be sorry if the podcast I regularly listen to were to shut</td><td>Adjusted wording for context</td><td>Retained</td></tr>
</table>

		down.				
**Perceived Social Presence (Lee, 2002; Gefen & Straub, 2003; Kang & Gretzel, 2012)**	1. I felt as if the/each narrator was talking to me.	—	No modification	Retained	—	Cronbach's α = .777; KMO = .833; Bartlett's test p < .001; CFA χ^2/df = 1.69, GFI = .986, AGFI = .967, RMSEA = .044, CFI = .988, TLI = .979
	2. I felt the narrator(s) conveyed feelings and emotions.	—	No modification	Retained		
	3. I was able to mentally imagine the/each narrator.	—	No modification	Retained		
	4. I felt cared for in the park even though there was no human guide.	4. While listening to the podcast, I feel cared for even in the absence of face-to-face interaction with the host.	Adapted for relevance to podcast listening context.	Retained		
	5. I felt involved with the narrator(s).	—	No modification	Retained		
	6. I perceived the narrator(s)' messages as	—	No modification	Retained		

	being personal.					
**Perceived Social Support (Chen & Keng, 2023)**	1.When faced with difficulties, some people in the podcast community have comforted and encouraged me.	—	Considered more relevant to highly interactive communities; less aligned with study objectives.	Removed	Cronbach's α = .845; KMO = .776; Bartlett's test p < .001; CFA: χ^2/df = 2.639, GFI = .979, AGFI = .945, RMSEA = .068, CFI = .988, TLI = .978	Cronbach's α = .843; KMO = .737; Bartlett's test p < .001; CFA: χ^2/df = 3.00, GFI = .987, AGFI = .950, RMSEA = .075, CFI = .992, TLI = .980
	2. When faced with difficulties, some people in the podcast community have listened to me and discussed my private feelings.	—	No modification	Retained		
	3. When faced with difficulties, some people in the podcast community sites have expressed interest and concern in my posts or messages.	—	No modification	Retained		

	4. My subscription to some podcast shows offers knowledge and suggestions when I need help.	—	No modification	Retained		
	5.When I encounter a problem, listening to some subscription podcast shows gives me information and knowledge to help me overcome the problem.	—	No modification	Retained		
	6. When faced with difficulties, listening to some subscription podcast shows helps me discover causes and gives	—	No modification	Retained		

	me knowledge and suggestions.					
**Subjective Well-Being (Suh & Koo, 2011)**	1. I am satisfied with the personal aspects of my life	—	No modification	Retained		Cronbach's α = .842; Life satisfaction α = .846; Positive affect α = .843; Negative affect α = .886; KMO = .811; Bartlett's test p < .001; CFA: χ^2/df = 2.807, GFI = .960, AGFI = .925, RMSEA = .071, CFI = .976, TLI = .964
	2. I am satisfied with the relational aspects of my life	—	No modification	Retained		
	3. I am satisfied with the collective aspects of my life	—	No modification	Retained		
	4. I experience moments of joy more frequently	—	No modification	Retained		
	5. I experience moments of happiness more frequently	—	No modification	Retained		
	6. I feel more peaceful in my state of mind	—	No modification	Retained		
	7. I feel angry more easily	—	No modification	Retained		
	8. I am more	—	No modification	Retained		

	prone to experiencing negative emotions					
	9. I feel helpless more easily	—	No modification	Retained		

Note: note: Analyses for the "Initial Item Pool" were conducted on data collected for this study, using the version containing all proposed modified items. The "Final Scale" results are based on the final version of the scale after removing items deemed unsuitable or psychometrically problematic, and represent the version used in subsequent analysis.
