# Supplementary material for: Podcast Listening, Perceived Social Presence, Perceived Social Support, and Subjective Well-Being Among Chinese Young Adults: Sequential Explanatory Mixed Methods Study
Source: Behav Sci (Basel). 2026 Feb 11;16(2):267. doi: 10.3390/bs16020267 (PMC12938595; doi:10.3390/bs16020267)
Supplement: Supplementary file 1 [file behavsci-16-00267-s001.zip › Supplementary File S2.pdf]

Supplementary File S2: Survey Questionnaire

Questionnaire on the Relationship Between Podcast Listening and Subjective Well-being Among Young People

Hello! First, thank you for participating in my survey! Second, your responses will be used for research purposes only and will be kept strictly confidential. You do not need to provide your name. Please answer according to your true situation (check the box √). This questionnaire investigates the relationship between podcast listening habits and subjective well-being. Please answer every item and do not leave any blank. There are no right or wrong answers, and no value judgments are involved.

The above information includes a statement of informed consent. Your completion of the questionnaire indicates that you have read and agreed to participate. If you confirm that you understand the content and purpose of this research survey and agree to participate, please fill out this questionnaire according to your true situation. If you decline to participate, please do not fill out the questionnaire.

1. Your Gender:

○ Male
○ Female

2. Your Age:

○ 16-18 years old
○ 19-22 years old
○ 23-26 years old
○ 27-30 years old
○ 31-35 years old

3. Your Education Level:

○ Junior high school or below

○ High school / Vocational school
○ Associate degree
○ Bachelor's degree
○ Master's degree
○ Doctoral degree

4. Your Marital Status:
○ Single
○ Married

5. Your Occupation:
○ Student
○ Employed
○ Freelancer / Self-employed
○ Full-time homemaker / Househusband
○ Other

6. Your Income Level:
○ Below 3,000 RMB
○ 3,001 - 5,000 RMB
○ 5,001 - 10,000 RMB
○ 10,000 RMB and above

7. How long have you been listening to podcasts?
○ Less than 6 months
○ 6 months to 1 year

○ 1-2 years
○ 2-3 years
○ 3-4 years
○ More than 4 years

8. How much time do you spend listening to podcasts per day?
○ Less than 0.5 hours
○ 0.5 - 1 hour
○ 1 - 2 hours
○ 2 - 4 hours
○ More than 4 hours

9. What categories of podcasts do you listen to? [Multiple Choice]
☐ Fashion & Beauty
☐ Fitness & Wellness
☐ Parenting & Family
☐ Workplace
☐ Self-improvement
☐ News
☐ Business
☐ Emotional Life / Relationships
☐ Technology
☐ Sports
☐ Leisure, Entertainment & Hobbies (Food, Gaming, Celebrity Gossip)
☐ Society, Culture & History
☐ Music, Film & TV, Books

☐ Comedy / Talk Shows
☐ Art
☐ Religion
☐ Mystery (True Crime, Supernatural Stories, Urban Legends)

10.To what extent do the following statements describe your podcast listening habits? Please select the most appropriate answer for each statement based on your judgment.

Statements	Strongly Disagree	Disagree	Neutral	Agree	Strongly Agree
Podcast is part of my everyday activity	o	o	o	o	o
Podcast has become part of my daily routine	o	o	o	o	o
I feel I am part of the podcast community	o	o	o	o	o
I would be sorry if the podcast I regularly listen to were to shut down.	o	o	o	o	o

11. To what extent do the following statements describe your feelings while listening to podcasts? Please select the most appropriate answer for each statement based on your judgment.

Statements	Strongly Disagree	Disagree	Neutral	Agree	Strongly Agree
I felt as if the/each narrator was talking to me	o	o	o	o	o
I felt the narrator(s) conveyed feelings and emotions	o	o	o	o	o
I was able to mentally imagine the/each narrator	o	o	o	o	o
While listening to the podcast, I feel cared for	o	o	o	o	o

Statements	Strongly Disagree	Disagree	Neutral	Agree	Strongly Agree
even in the absence of face-to-face interaction with the host					
I felt involved with the narrator(s)	○	○	○	○	○
I perceived the narrator(s)' messages as being personal	○	○	○	○	○

12.To what extent do the following statements describe your feelings regarding podcast listening? Please select the most appropriate answer for each statement based on your judgment.

Statements	Strongly Disagree	Disagree	Somewhat Disagree	Neutral	Somewhat Agree	Agree	Strongly Agree
When faced with difficulties, some people in the podcast community have listened to me and discussed my private feelings	○	○	○	○	○	○	○
When faced with difficulties, some people in the podcast community have expressed interest and concern in my posts or messages	○	○	○	○	○	○	○
My subscription to some podcast shows offers	○	○	○	○	○	○	○

Statements	Strongly Disagree	Disagree	Somewhat Disagree	Neutral	Somewhat Agree	Agree	Strongly Agree
knowledge and suggestions when I need help							
When I encounter a problem, listening to some subscription podcast shows gives me information and knowledge to help me overcome the problem	o	o	o	o	o	o	o
When faced with difficulties, listening to some subscription podcast shows helps me discover causes and gives me knowledge and suggestions	o	o	o	o	o	o	o

13. Please recall your feelings since you started listening to podcasts. To what extent do the following statements describe your experience?

Statements	Strongly Disagree	Disagree	Somewhat Disagree	Neutral	Somewhat Agree	Agree	Very Strongly Agree
I am satisfied with the personal aspects of my life	o	o	o	o	o	o	o
I am satisfied with the relational aspects of my life	o	o	o	o	o	o	o
I am satisfied with the collective aspects of my life	o	o	o	o	o	o	o

Statements	Strongly Disagree	Disagree	Somewhat Disagree	Neutral	Somewhat Agree	Agree	Very Strongly Agree
I experience moments of joy more frequently	o	o	o	o	o	o	o
I experience moments of happiness more frequently	o	o	o	o	o	o	o
I feel more peaceful in my state of mind	o	o	o	o	o	o	o
I feel angry more easily	o	o	o	o	o	o	o
I am more prone to experiencing negative emotions	o	o	o	o	o	o	o
I feel helpless more easily	o	o	o	o	o	o	o
