# Supplementary material for: Podcast Listening, Perceived Social Presence, Perceived Social Support, and Subjective Well-Being Among Chinese Young Adults: Sequential Explanatory Mixed Methods Study
Source: Behav Sci (Basel). 2026 Feb 11;16(2):267. doi: 10.3390/bs16020267 (PMC12938595; doi:10.3390/bs16020267)
Supplement: Supplementary file 1 [file behavsci-16-00267-s001.zip › Supplementary File S3.pdf]

Supplementary File S3: Participant Information

ID	Gender	Age	Education Level	Occupation	Daily Listening Time	Listening Duration	Preferred Podcast Genres	Subjective Well-Being Score
P01	Female	23	Master's degree	Employed	5 h/day	2 years	Fitness & Wellness | Workplace | Self-improvement | News | Society, Culture & History | Music, Film & TV, Books | Comedy / Talk Shows | Arts	5.56
P02	Male	25	Bachelor's degree	Student	1 h/day	6 months	Workplace | Self-improvement | Business | Technology	5.33
P03	Male	26	Bachelor's degree	Employed	3 h/day	3 years	Fitness & wellness | Workplace | News | Leisure, Entertainment & Hobbies (Food, Gaming, Celebrity Gossip) | Society, Culture & History | Music, Film & TV, Books | Comedy / Talk Shows | Mystery (True Crime, Supernatural Stories, Urban Legends)	3.67
P04	Male	22	Bachelor's degree	Student	1 h/day	2 years	Leisure, Entertainment & Hobbies (Food, Gaming, Celebrity Gossip) | Music, Film & TV, Books | Comedy / Talk Shows | Mystery (True Crime, Supernatural Stories, Urban Legends)	3.67
P05	Female	26	High school	Employed	2 h/day	3 years	Fashion & beauty | Parenting & family | Self-improvement | Music, Film & TV, Books | Comedy / Talk Shows | Mystery (True Crime, Supernatural Stories, Urban Legends)	5.56
P06	Female	22	Master's degree	Student	0.5 h/day	2 years	Self-improvement | Business | Comedy / Talk Shows	5.78
P07	Female	21	Bachelor's degree	Student	0.5 h/day	6 months	Emotional life | Leisure, Entertainment & Hobbies (Food, Gaming, Celebrity Gossip) | Music, Film & TV,	3.89

							Books | Comedy / Talk Shows	
P08	Female	22	High school	Employed	2 h/day	1 year	Workplace | Self-improvement | Emotional life | Leisure, Entertainment & Hobbies (Food, Gaming, Celebrity Gossip)	3.89
P09	Male	33	Associate degree	Employed	2 h/day	4 years	Fitness & wellness | Self-improvement | Emotional life | Technology | Leisure, Entertainment & Hobbies (Food, Gaming, Celebrity Gossip) | Music, Film & TV, Books	3.79
P10	Female	30	Master's degree	Employed	1 h/day	2 years	Leisure, Entertainment & Hobbies (Food, Gaming, Celebrity Gossip) | Society, Culture & History | Music, Film & TV, Books | Comedy / Talk Shows | Arts | Religion | Mystery (True Crime, Supernatural Stories, Urban Legends)	3.79
P11	Female	19	Bachelor's degree	Freelancer	1 h/day	1 year	Fashion & beauty | Fitness & Wellness | Parenting & family | Workplace | Self-improvement | News | Business | Emotional life | Technology | Sports | Leisure, Entertainment & Hobbies (Food, Gaming, Celebrity Gossip) | Society, Culture & History | Music, Film & TV, Books | Comedy / Talk Shows | Arts | Mystery (True Crime, Supernatural Stories, Urban Legends)	5.67
P12	Male	24	Bachelor's degree	Employed	2 h/day	3 years	Self-improvement | Technology | Leisure, Entertainment & Hobbies (Food, Gaming, Celebrity Gossip) | Society, Culture & History | Music, Film & TV, Books | Mystery (True Crime, Supernatural Stories, Urban Legends)	5.33

P13	Female	26	Master's degree	Student	0.5 h/day	6 months	Self-improvement	4.00
P14	Female	20	Bachelor's degree	Student	1 h/day	2 years	Workplace | Self-improvement | Leisure, Entertainment & Hobbies (Food, Gaming, Celebrity Gossip) | Music, Film & TV, Books | Mystery (True Crime, Supernatural Stories, Urban Legends)	5.67
P15	Female	25	Bachelor's degree	Employed	1 h/day	2 years	Workplace | Self-improvement	4.00
P16	Male	28	Associate degree	Employed	3 h/day	3 years	Fitness & Wellness | Workplace | News | Business | Emotional life	4.00
P17	Male	23	Bachelor's degree	Employed	3 h/day	4 years	Leisure, Entertainment & Hobbies (Food, Gaming, Celebrity Gossip) | Society, Culture & History	5.44
P18	Male	25	Bachelor's degree	Employed	2 h/day	4 years	News | Business | Emotional life | Leisure, Entertainment & Hobbies (Food, Gaming, Celebrity Gossip) | Society, Culture & History	5.44
P19	Female	32	Doctoral degree	Employed	4 h/day	5 years	Self-improvement | Emotional life | Leisure, Entertainment & Hobbies (Food, Gaming, Celebrity Gossip)	5.56
P20	Female	20	Bachelor's degree	Student	1 h/day	1 year	Self-improvement | Leisure, Entertainment & Hobbies (Food, Gaming, Celebrity Gossip) | Comedy / Talk Shows | Mystery (True Crime, Supernatural Stories, Urban Legends)	3.79
