# Supplementary material for: Podcast Listening, Perceived Social Presence, Perceived Social Support, and Subjective Well-Being Among Chinese Young Adults: Sequential Explanatory Mixed Methods Study
Source: Behav Sci (Basel). 2026 Feb 11;16(2):267. doi: 10.3390/bs16020267 (PMC12938595; doi:10.3390/bs16020267)
Supplement: Supplementary file 1 [file behavsci-16-00267-s001.zip › Supplementary File S4.pdf]

Supplementary File S4: Interview Guide

1. In what situations do you usually think about listening to podcasts? What typically motivates you to start listening?
2. What role do podcasts play in your everyday life, particularly when you are under stress or feeling emotionally strained?
3. Compared with other media formats (e.g., long-form video, short videos, or music), what do you perceive as distinctive about podcasts?
4. Are there any moments or periods when podcasts have felt particularly important in your life? If so, could you describe them?
5. Have there been any episodes in which you felt that the host was “present” or as if they were “speaking directly to you”?
6. When you experience a sense that the podcast host is “accompanying” you, what do you think this feeling stems from? For example, does it relate to the host’s voice, tone, topics, or narrative style?
7. While listening to podcasts, do you ever find yourself responding to the host internally (e.g., nodding, smiling, disagreeing, or mentally replying)? How do you think this sense of “interaction” emerges?
8. If you stop listening to podcasts for a period of time, do you find yourself missing certain hosts or programs? Why or why not?
9. Have there been moments when listening to a podcast made you feel “understood” or that the host “articulated something you had been feeling”? What contributed to that experience?
10. When podcast hosts share their own experiences or emotions, do you feel a sense of resonance or companionship-based support? Could you elaborate?
11. Do you think podcasts have provided you with emotional comfort or practical advice in any way? Please give examples if possible.
12. Have there been times when listening to podcasts noticeably improved your mood? Could you describe a specific situation?
13. How does the sense of support derived from podcasts influence your life, emotional state, stress levels, or overall well-being?
14. Do you consider podcast listening to be a way of enhancing subjective well-being? Why or why not?
15. Do the positive experiences gained from podcasts carry over into your offline relationships, life attitudes, or self-perception? If so, how?
16. Overall, what do you see as the most important significance of podcasts in your life?
17. Is there anything else you would like to share about your podcast listening experiences, stories, or feelings?
