# Supplementary material for: Podcast Listening, Perceived Social Presence, Perceived Social Support, and Subjective Well-Being Among Chinese Young Adults: Sequential Explanatory Mixed Methods Study
Source: Behav Sci (Basel). 2026 Feb 11;16(2):267. doi: 10.3390/bs16020267 (PMC12938595; doi:10.3390/bs16020267)
Supplement: Supplementary file 1 [file behavsci-16-00267-s001.zip › Supplementary File S5.pdf]

Supplementary File S5: Robustness Check

Path and Effect	Model A: Baseline Model (Controlling for Demographics)	Model B: Stringent Model (Additionally Controlling for Podcast-Use Characteristics)
Regression Paths (Unstandardized Coefficient B)		
PL → PSP	0.405***	0.385***
PL → PSS	0.202**	0.152
PSP → PSS	0.936***	0.901***
PSP → SWB	0.300**	0.323***
PSS → SWB	0.144**	0.122*
PL → SWB (Direct Effect c')	0.029	0.001
Bootstrap Indirect Effects [95% CI]		
Total Indirect Effect	0.205 [.130, .294]	0.185 [.113, .276]
Specific Indirect Effect 1: PL → PSP → SWB	0.122 [.039, .212]	0.124 [.044, .216]
Specific Indirect Effect 2: PL → PSS → SWB	0.029 [.000, .078]	0.019 [-.003, .061]
Specific Indirect Effect 3: PL → PSP → PSS → SWB	0.055 [.013, .100]	0.042 [.004, .091]

note: N = 357. *p < .05, **p < .01, ***p < .001. Bootstrap confidence intervals are bias-corrected. Model A controls for age, gender, and education. Model B adds controls for podcast listening frequency, duration, and device type.

Table S1. Comparison of path coefficients across the original serial mediation model and alternative model specifications.

Path	Original model (β/SE/t/p)	Model1 (b/SE/t/p)	Model 2 (b/SE/t/p)
PL → PSS/PSP	PL → PSP: 0.492*** (SE=0.046, t=10.63)	PL → PSP: 0.580*** (SE=0.076, t=7.69, CI[0.43,0.73])	SWB → PSP: 0.231*** (SE=0.035, t=6.55, CI[0.16,0.30])
PL → PSP/PSS	PL → PSS: 0.129** (SE=0.049, t=2.63)	PL → PSS: 0.268*** (SE=0.036, t=7.40, CI[0.20,0.34])	SWB → PSS: 0.193** (SE=0.060, t=3.20, CI[0.07,0.31])
PSS/PSP →	PSP → PSS: 0.492*** (SE=0.049,	PSS → PSP: 0.236*** (SE=0.024,	PSP → PSS: 0.967*** (SE=0.086,

PSP/PSS	t=9.97)	t=9.97, CI[0.19,0.28])	t=11.24, CI[0.80,1.14])
PL → SWB (direct)	PL → SWB: 0.024 (ns, SE=0.057, t=0.43, CI[-0.09,0.14])	PL → SWB: 0.029 (ns, SE=0.067, t=0.43, p=0.67, CI[-0.10,0.16])	SWB → PL: 0.018 (ns, SE=0.042, t=0.43, p=0.67, CI[-0.07,0.10])
PSP/PSS → SWB	PSS → SWB: 0.191** (SE=0.062, t=3.10)	PSS → SWB: 0.300** (SE=0.092, t=3.25, p=0.001, CI[0.12,0.48])	PSP → PL: 0.498*** (SE=0.070, t=7.17, CI[0.36,0.64])
PSS/PSP → SWB	PSP → SWB: 0.210** (SE=0.065, t=3.25)	PSP → SWB: 0.144** (SE=0.046, t=3.10, p=0.002, CI[0.05,0.24])	PSS → PL: 0.093* (SE=0.037, t=2.52, p=0.012, CI[0.02,0.17])
Covariates	Covariates→ PSP: -0.164***; → PSS: -0.053 (ns); → SWB: -0.080 (ns)	Covariates→ PSP: -0.167** (p=0.009); → PSS: -0.069* (p=0.016); → SWB: -0.076 (ns)	Covariates → PSP: -0.073* (p=0.036); → PSS: -0.036 (ns); → PL: 0.080* (p=0.041)

Table S2. Decomposition of total, direct, and indirect effects across the original model and alternative model specifications.

**Effect Type**	**Original model (βcs / BootSE / CI / % of Total Effect)**	**model 1 (βcs / BootSE / CI / % of Total Effect)**	**model 2 (βcs / BootSE / CI / % of Total Effect)**
Direct Effect (c')	0.024 (ns, 0.057, [-0.088, 0.137], 12.1%)	0.029 (ns, 0.076, [-0.123, 0.172], ≈14%)	0.018 (ns, 0.048, [-0.072, 0.117], ≈10%)
Indirect Effect	0.174*** (0.035, [0.114, 0.250], 87.9%)	0.205*** (0.042, [0.131, 0.296], 87.7%)	0.154*** (0.030, [0.100, 0.219], 89.5%)
Ind1	PL → PSP → SWB: 0.103** (0.038, [0.033, 0.184], 52.0%)	PL → PSS → SWB: 0.084** (0.037, [0.019, 0.164], 36.0%)	SWB → PSP → PL: 0.115*** (0.027, [0.067, 0.175], 67.0%)
Ind2	PL → PSS → SWB: 0.025* (0.017, [0.001, 0.068], 12.6%)	PL → PSP→ SWB: 0.080** (0.032, [0.025, 0.149], 34.2%)	SWB → PSS → PL: 0.018 (ns, 0.013, [-0.001, 0.049], 10.5%)
Ind3	PL → PSP → PSS → SWB: 0.046** (0.019, [0.013, 0.087], 23.2%)	PL → PSS → PSP → SWB: 0.041** (0.016, [0.013, 0.075], 17.5%)	SWB → PSP → PSS → PL: 0.021* (0.011, [0.001, 0.042], 12.2%)

Table S3. Comparison of explained variance and model fit across the original and alternative model specifications.

Model	PSP Equation (R^2 / F)	PSS Equation (R^2 / F)	SWB Equation (R^2 / F)
Original Model	PSP: 0.263***, F(4,352)=31.46	PSS: 0.371***, F(5,351)=41.38	SWB: 0.164***, F(5,351)=11.41
Model 1	PSP: 0.193***, F(4,352)=21.01	PSS: 0.426***, F(5,351)=52.07	SWB: 0.164***, F(6,350)=11.41
Model 2	PSP: 0.131***, F(4,352)=13.21	PSS : 0.377***, F(5,351)=42.41	PL: 0.268***, F(6,350)=21.37

note: Model 1: PL → PSS → PSP → SWB; Model 2: SWB → PSP → PSS → PL
