# Supplementary material for: Podcast Listening, Perceived Social Presence, Perceived Social Support, and Subjective Well-Being Among Chinese Young Adults: Sequential Explanatory Mixed Methods Study
Source: Behav Sci (Basel). 2026 Feb 11;16(2):267. doi: 10.3390/bs16020267 (PMC12938595; doi:10.3390/bs16020267)
Supplement: Supplementary file 1 [file behavsci-16-00267-s001.zip › Supplementary File S6.pdf]

Supplementary File S6: Details of Coding

**Code**	**Definition**	**Illustrative Quote**
Theme 1. Experiencing Social Presence in Podcast Listening		
1.1 Ambient Companionship	Podcasts function as low-threshold background audio embedded in everyday routines, providing a continuous sense of companionship during solitary activities.	*"I usually listen to podcasts during my commute, while doing my makeup, or whenever I'm feeling a bit down."(P05)*
1.2 Temporal Anchoring	Podcast listening becomes routinized at specific times of day, helping listeners structure time.	*"I usually turn to podcasts proactively when I find myself tossing and turning before bed—they have become my preferred way to unwind and fall asleep."(P08)*
1.3 Immersion Evoked by Voice	Listeners experience social presence through vocal cues such as tone, rhythm, pauses, and emotional expressiveness, which create intimacy and psychological closeness.	*"When I feel immersed in a podcast host's delivery, it hinges on their voice, tone, and the sincerity of their content. A soft, rich voice naturally conveys warmth and approachability, instantly closing the distance. A steady, composed tone—free from artificiality or overacting—puts listeners at ease, as if they're part of a casual conversation."(P01)*
1.4 Pseudo-social Interaction	Listeners engage in internal cognitive or emotional responses (e.g., nodding, laughing, mentally replying), forming a one-way yet immersive interaction with the host.	*"I often find myself responding to the host's opinions in my head. When they talk about something funny, I feel happy along with them; when they share something sad, I might even tear up. This sense of interaction comes mainly from being fully immersed in what they're talking about and emotionally present in the moment they're creating."(P05)*
Theme 2. Perceived Social Support in Podcast Listening		
2.1 Perceived Emotional	Podcasts evoke feelings of being understood or acknowledged when hosts articulate emotions or experiences that resonate with listeners' personal	*"I was feeling pretty low for a while, and an episode where Lu Yu talked with Zhang Chun really gave me a lot of strength. There was also an episode from a smaller show about "regaining vitality" that I*

Support	struggles.	*found really inspiring. It got me moving again and doing small things, like going to the park on the weekend or doing some bead crafts. I've gained a lot of energy from these little moments."(P04)*
2.2 Perceived Informational Support	Podcasts provide practical knowledge, experiences, or cognitive frameworks that help listeners interpret situations and make decisions.	*"Some podcasts even give tips on sleep positions. I tried one, and it really seemed to help me sleep more soundly."(P17)*
2.3 Imagined and Enacted Community Support	A sense of support derived from awareness of a shared audience or participation in podcast-related communities, even without direct interaction.	*" We also have a listener community where people discuss the show and actively contribute ideas."(P11)*
Theme 3. Sequential Experiences of Podcast Listening		
3.1 Stabilization of Social Presence	Repeated listening transforms the host's voice into a familiar and enduring presence rather than a novel auditory stimulus.	*" I've long thought of my favorite podcasters as friends I've never met but feel deeply connected to—their thoughts and values always resonate with mine. Going too long without listening feels like losing touch with an old friend. I find myself wondering how they've been, eager to hear their latest insights and reflections. I miss the energy I gain from their shared experiences, and this concern for my "friends'" well-being always draws me back to catch up on episodes I've missed."(P01)*
3.2 Perceived Relevance and Emotional Relief	When podcast content aligns with listeners' current concerns, it facilitates emotional relief and cognitive reframing.	*"I think there are. For example, I once listened to a podcast where the host talked about peer pressure. At that time, I was feeling pretty down because of peer pressure, so I was kind of stuck in a gloomy mood. But after listening to that episode, I got a new perspective on the topic and on my own situation. I felt comforted by it. "(P15)*

3.3 Temporally Unfolding Presence–Support Process	Social presence, as a media-mediated experience, gradually evolves into a stable form of perceived social support through repeated and emotionally resonant listening.	*“When the host shared experiences that mirrored my own, it made the world feel truly wonderful—someone else out there was feeling exactly what I did. Hearing their stories reminded me that I wasn't alone in facing the same challenges. Their experiences offered reassurance: don't panic; perhaps all I need is just a little more effort.”(P13)*
3.4 Multidimensional Subjective Well-Being	This theme refers to participants’ perceived enhancement of subjective well-being across multiple dimensions, including increased positive emotions (e.g., optimism and happiness), greater life satisfaction, and a reduction in negative emotional states such as stress and emotional tension. Notably, these feelings of well-being extend beyond listening contexts and carry over into participants’ offline lives.	*“Listening to podcasts makes me reflect on some of the problems I have in my relationships. It helps me cut down on overthinking and anxiety, so I feel more optimistic about life and better able to deal with complicated social situations. Overall, it puts me in a better headspace and makes me feel happier and more positive.”(P05)*
Theme 4. Additional Experiential Patterns Beyond the Serial Mediation Model		
4.1 High Social Presence Without Perceived Support	Listeners experience strong companionship and immersion without interpreting it as emotional or instrumental support.	*“When I listen to podcasts, I rarely feel “understood” or like I’m voicing my inner thoughts. What draws me most is the host’s unique perspective. For me, the real value of podcasts lies in expanding my cognitive horizons. When a host interprets workplace rules or life phenomena from a completely new angle, or shares ideas I’ve never encountered before, it feels fresh and fascinating. This sense of novelty pushes me to let go of my preconceptions and embrace new ways of thinking. It not only enriches my own thought process but also helps me see the world from more diverse perspectives—an experience far more meaningful than simply being understood.”(P18)*

4.2 Boundary Conditions of Support Formation	The transformation of social presence into perceived social support depends on content relevance and listeners' interpretive frames.	*“I think the most important role of podcasts in my life is that they offer an additional perspective in a fairly relaxed way. For me, podcasts are still more of a channel for taking in knowledge and information, rather than purely for entertainment or for emotional release and stress relief.”(P14)*
